# A Review of the Toxicity and Phytochemistry of Medicinal Plant Species Used by Herbalists in Treating People Living With HIV/AIDS in Uganda

**DOI:** 10.3389/fphar.2021.615147

**Published:** 2021-04-15

**Authors:** G. Anywar, E. Kakudidi, R. Byamukama, J. Mukonzo, A. Schubert, H. Oryem-Origa, C. Jassoy

**Affiliations:** ^1^ Department of Plant Sciences, Microbiology and Biotechnology, College of Natural Sciences, Makerere University, Kampala, Uganda; ^2^ Fraunhofer Institute for Cell Therapy and Immunology (IZI), Leipzig, Germany; ^3^ Institute for Virology, University Clinics and Faculty of Medicine, University of Leipzig, Leipzig, Germany; ^4^ Department of Chemistry, College of Natural Sciences, Makerere University, Kampala, Uganda; ^5^ Department of Pharmacology and Therapeutics, College of Health Sciences, Makerere University, Kampala, Uganda

**Keywords:** phytochemistry, medicinal plants, herbalists, antiviral, hiv/aids, Uganda, toxicity

## Abstract

**Introduction:** Despite concerns about toxicity, potentially harmful effects and herb-drug interactions, the use of herbal medicines remains widely practiced by people living with HIV/AIDS (PLHIV) in Uganda.

**Objective:** The objective of the paper was to comprehensively review the literature on the toxicity and chemical composition of commonly used medicinal plant species in treating PLHIV in Uganda.

**Methods:** We reviewed relevant articles and books published over the last sixty years on ethnobotany, antiviral/anti-HIV activity, toxicity, phytochemistry of *Vachellia hockii*, *Albizia coriaria*, *Bridelia micrantha*, *Cryptolepis sanguinolenta*, *Erythrina abyssinica*, *Gardenia ternifolia*, *Gymnosporia senegalensis*, *Psorospermum febrifugium*, *Securidaca longipendunculata*, *Warburgia ugandensis* and *Zanthoxylum chalybeum* and their synonyms. We searched PubMed, Web of Science, Scopus, Science Direct and Google Scholar.

**Discussion:** Most of the plant species reviewed apart from *P. febrifugium*, *S. longipedunculata* and *C. sanguinolenta* lacked detailed phytochemical analyses as well as the quantification and characterization of their constituents. Crude plant extracts were the most commonly used. However, purified/single component extracts from different plant parts were also used in some studies. The U87 human glioblastoma was the most commonly used cell line. Water, ethanol, methanol and DMSO were the commonest solvents used. In some instances, isolated purified compounds/extracts such as Cryptolepine and Psorospermin were used.

**Conclusion:** Cytotoxicity varied with cell type, solvent and extract type used making it difficult for direct comparison of the plant species. Five of the eleven plant species namely, *A. coriaria*, *C. sanguinolenta*, *G. ternifolia*, *P. febrifugium* and *Z. chalybeum* had no cytotoxicity studies in animal models. For the remaining six plant species, the crude aqueous and ethanol extracts were mainly used in acute oral toxicity studies in mice. Herbalists reported only *A. coriaria* and *W. ugandensis* to cause toxic side effects in humans. However, selective cytotoxic plant extracts can potentially be beneficial as anticancer or anti-tumour drugs.

## Introduction

Traditional herbal medicines from medicinal plants are widely used among people living with HIV/AIDS (PLHIV) in Uganda (Anywar et al., 2020a; Anywar et al., 2020b). Although medicinal plants are generally considered to be safe, they are not entirely free of side effects or toxicity (Boukandou Mounanga et al., 2015). The toxicity of medicinal plants varies with chemical composition of the plant in question. The potential toxicity of traditional herbal medicines can arise from acute or chronic exposure even with extracts of low toxicity. The plant extracts could also be mutagenic or carcinogenic (Ferreira-Machado et al., 2004). Despite the benefits derived from medicinal plants, some may be a threat to the health of the users, due to potential harmful effects or side effects which may be related to overdoses or toxic principles. This may lead to acute toxicity and death of patients (Tamokou and Kuete, 2014; Schultz et al., 2020).

The use of traditional herbal medicines is associated with dysfunctions of the liver and kidneys in humans. Several animal studies have also shown some medicinal plants to be toxic to different organs. Toxic plants can affect a whole range of organ systems, with some plants having several toxic principles affecting multiple systems (Tamokou and Kuete, 2014). For instance, the medicinal plant *Aphania* *senegalensis* is hepatotoxic in rats (Fall et al., 2011) and *Herniaria cinerea* is toxic for the gastrointestinal tract (Sokar et al., 2003). However, the identities of toxic substances in African herbal medicines, their toxicology and pathogenesis are largely unknown (Luyckx et al., 2002). The objective of this paper was to comprehensively review the literature on the toxicity and phytochemistry of commonly used medicinal plant species in treating PLHIV in Uganda, to establish any correlation between traditional use, phytochemistry and toxicity.

## Methods

We reviewed relevant published articles and books from 1960 to date, covering a period of sixty years on ethnobotany, pharmacological or biological activity, toxicity, phytochemistry, of selected medicinal plant species used by herbalists in treating people living with HIV/AIDS in Uganda. We searched PubMed, Web of Science, Scopus, Science Direct and Google Scholar using search terms as ethnobotany, toxicity, cytotoxicity, phytochemistry, traditional use.

Eleven commonly used plant species for treating PLHIV in Uganda were chosen from a previous ethnobotanical study in Uganda namely; *Vachellia hockii* (De Wild.) Seigler and Ebinger., *Albizia coriaria* Welw. ex Oliv., *Bridelia micrantha* (Hochst.) Baill., *Cryptolepis sanguinolenta* (Lindl.) Schltr., *Erythrina abyssinica* Lam., *Gardenia ternifolia* Schumach. and Thonn., *Gymnosporia senegalensis* (Lam.) Loes., *Psorospermum febrifugium* Spach, *Securidaca longipedunculata* Fresen., *Warburgia ugandensis* Sprague, *Zanthoxylum chalybeum* Engl. The species were chosen because of their popularity and high frequently of use and citation in the study (Anywar et al., 2020a). Some of these plant species have variable anti- HIV effects (Bessong et al., 2006). Where the full botanical taxonomic names from the original studies were synonyms or inconsistent with the fully taxonomically validated names, the correct fully taxonomically validated species names have been applied in this review, using the Kew Medicinal Plant Names Services (MPNS), at https://mpns.science.kew.org/mpns-portal/, accessed on 16th December 2020.

## Discussion

### Toxicity and Cytotoxicity of Medicinal Plant Species

The toxicity of the plant extracts was reviewed at cellular and whole organism level. We discuss both *in vitro* and *in vivo* toxicity of the plant species as well as their phytochemical composition. According to Horvath (Horvath, 1980), cytotoxic compounds prevent cellular attachment, cause dramatic morphological changes, adversely affect replication rates, or lead to a reduced overall viability. However, cytotoxicity is dependent on the length of time of exposure of the cells to the compounds, mechanism of cytotoxicity and the kind of compound under test (Riss and Moravec, 2004; Di Nunzio et al., 2017). The toxicity and chemical composition of each of the eleven plant species are discussed in detail as follows.

#### *Albizia coriaria* Oliv.

*A. coriaria* is widely used in traditional medicine for treating opportunistic infections among PLHIV. *A. coriaria* is particularly used for skin infections, cough, sore throats, syphilis, jaundice in Uganda (Anywar et al., 2020a; Schultz et al., 2020 ; Namukobe et al., 2011), sexually transmitted infections, cancer (Schultz et al., 2020). The methanol and aqueous extracts of *A. coriaria* have very low cytotoxicity (CC_50_ > 500 μg/ml) against human embryonic lung fibroblast cells (HELF) (Kigoundu et al., 2009). The methanol, ethanol, ethyl acetate and diethyl ether extracts of *A. coriaria* were not toxic to human keratinocyte cell line (HaCaT) IC_50_ > 512 (Maroyi, 2017). *Albizia* spp. contained a toxic compound, 4-Methoxypyridoxine that is a vitamin B6 antagonist (Botha and Penrith, 2008). (Byamukama et al., 2015; Osebe et al., 2016) conducted a phytochemical evaluation of the ethyl acetate extract of *A. coriaria* extracts and identified and characterized lupeol, lupenone, betulinic acid, acacic acid lactone, (+)—Catechin and Benzyl alcohol. Anywar et al. documented prolonged boiling of *A. coriaria* decoctions for up to 6 h and administering it in small amounts by herbalists in Uganda (Anywar et al., 2020a). This is because decoctions of *A. coriaria* are known to have frequent side effects such as vomiting, dizziness, weakness when not properly well prepared and administered orally. *A. coriaria* extracts were contraindicated in pregnancy and in weak patients (Anywar et al., 2020a; Anywar et al., 2020b; Anywar, 2020). From the studies reviewed, there is conflicting information on the toxicity of *A. coriaria* species. Two studies indicate low to no cytotoxicity whereas one study points to high toxicity of *A. coriaria*. The high toxicity reported in traditional medicine is supported by a single cytotoxicity study (Anywar, 2020) and observations of poisonings in cattle (Botha and Penrith, 2008). The observations on toxicity support the practice of herbalists boiling the herb for long, administering low doses, mixing it with other herbs and not giving it to pregnant women. Further studies are needed on the acute and chronic toxicity of *A. coriaria* as well more cytotoxicity studies using different cell lines. It is also paramount that its anti-HIV activity is investigated to justify its popular use in HIV treatment.

#### *Bridelia micrantha* (Hochst.) Baill.

Maroyi (2017) conducted an extensive review on *B. micrantha* and showed that it is traditionally used across many parts of Africa for treating various ailments including gastric ulcers, sexually transmitted infections and coughs. *B. micrantha* is widely used in treating a variety of opportunistic infections among PLHIV in Uganda (Anywar et al., 2020a).

The cytotoxicity and toxicity of *B. micrantha* has been investigated using different human cell lines, brine shrimp and animal models. The ethanol and DMSO extracts were slightly less toxic to U87 glioblastoma cells with CC_50_ values of 96.8 and 76.1 μg/ml respectively (Bessong et al., 2006). Steenkamp et al. showed the aqueous extract of *B. micrantha* to be cytotoxic against HeLa cells (IC_50_ = 8.9 μg/ml) and human breast cells (MCF-12A), IC_50_ = 24.2 μg/ml. Likewise, (Omosa et al., 2016) found the dichloromethane and methanol extracts of the leaves and stem bark of *B. micrantha* to be cytotoxic to CCRF-CEM leukaemia cells with IC_50_ values of 9.43 μg/ml and 23.5 μg/ml respectively. Using the brine shrimp lethality assay, (Osebe et al., 2016) showed the water stem bark extract of *B. micrantha* to have a 50% lethal concentration (LC_50_) of 77 μg/ml, and was considered toxic. Using the same assay, (Moshi et al., 2010) showed the ethanol root extract of *B. micrantha* to be mildly toxic, LC_50_ = 30 μg/ml. Oral administration of the stem bark extract of *B. micrantha* was well tolerated at a dose of 2000 mg/kg in male Wistar rats without any deaths or clinical signs of toxicity 48 h after administration (Onoja et al., 2014) found that *B. micrantha* contains several phytochemicals such as alkaloids, anthraquinones, cyanogenic glycoside, essential oils, esters, flavonoids, saponins, sterols, tannins, terpenoids and phenolics (Maroyi et al., 2017). Compounds isolated from *B. micrantha* include: friedelin, taraxerone, epifriedelinol, taraxerol, gallic acid, ellagic acid and -sitosterol (Bessong et al., 2006; Kouam et al., 2005; Pegel, 1968)

The n-butanol fraction from the crude methanol extracts of the roots of *B. micrantha* showed the highest RNA-dependent-DNA polymerization (RDDP) inhibitory activity of HIV-1 reverse transcriptase (RT) with an IC_50_ of 7.3 μg/ml (Bessong et al., 2006). This antiviral activity against RT with varying levels of cytotoxicity, depending on the cell-type used supports the traditional use of this plant in HIV treatment.

#### *Cryptolepis sanguinolenta* (Lindl.) Schltr

*C. sanguinolenta* is popularly used for treating malaria, bacterial respiratory diseases, hypertension, diarrhoea and urinogenital infections (Iwu 1993) skin infections (Anywar et al., 2020a). The aqueous root extract of *C. sanguinolenta* was cytotoxic to various human cancer cell lines using the resazurin alamar blue assay. The extract had IC_50_ values of 38.8 μg/ml against a Chinese hamster lung fibroblast cell line (V79-MZ), and the following human cancer cell lines: 1) 20.8 μg/ml against colon adenocarcinoma cell line (HCT-116), 2) 23.2 μg/ml against the ovary adenocarcinoma cell line (SKOV3), 3) 13.9 μg/ml against the MCF7, and 4) 68.8 μg/ml against the breast adenocarcinoma (MDA MB 361); thus demonstrating a wide variety of anticancer activity (Ansah and Gooderham, 2002). *C. sanguinolenta* contains the alkaloid cryptolepine and isocryptolepine (Grellier et al., 2002; Bonjean et al., 1998), polyuronides, anthocyanosides and triterpenes (Mills-Robertson et al., 2012). Cryptolepine is known to be a cytotoxic drug (Mills-Robertson et al., 2012). Cryptolepine has been shown to be cytotoxic to B16 melanoma cells (IC_50_ = 0.3 μg/ml) (Lisgarten et al., 2002). The aqueous extracts of *C. sanguinolenta* are potentially genotoxic as they act as a DNA intercalator. They also exhibit potent toxicity to a variety of mammalian cells *in vitro* (Ansah et al., 2005). However, cryptolepine has low cytotoxicity against normal human epidermal melanocytes and melanoma cell lines (Pal et al., 2017). Different doses of *C. sanguinolenta* extract caused a reduction in male fertility in mice, coupled with a reduced female fertility index (Ansah et al., 2010). The metabolism of cryptolepine in humans *in vitro* is similar to that in rats. The plasma clearence of cryptolepine is rapid with widespread distribution and modeate shelf life (Forkuo et al., 2017). Most of the work of *C. sanguinolenta* is focused on malaria treatment since malaria is an important cause of disease in PLHIV (Alemu et al., 2013). However, specific studies need to be conducted on the anti-HIV effects of *C. sanguinolenta*.

#### *Erythrina* abyssinica Lam.

*E. abyssinica* is used for treating syphilis, fevers and malaria (Alemu et al., 2013) tuberculosis (Ssegawa and Kasenene, 2007) and various opportunistic infections in HIV/AIDS (Anywar et al., 2020a). The crude alkaloidal fraction *E. abyssinica* caused a reduction in the viability of mock-infected MT-4 cells with a CC_50_ of 53 mM and a 50% protection of MT-4 cells against HIV-1 induced cytopathogeneticy with an EC_50_ of 453 mM (Mohammed et al., 2012). The *in vitro* cytotoxicity of the crude alkaloidal fraction of the seeds of *E. abyssinica* against HeLa, Hep-G2, HEP-2, HCT116, MCF-7 and HFB4 cell lines had IC_50_ values of 13.8, 10.1, 8.16, 13.9, 11.4 and 12.2 μg/ml, respectively (Mohammed et al., 2012). Anywar (2020) found the stem bark of *E. abyssinica* to be less toxic than seed extracts with IC_50_ values of 65.9 μg/ml for the crude ethanol extract and 72.9 μg/ml for the DMSO extract and concluded that *E. abyssinica* was moderately cytotoxic to U87.CD4.CXCR4 cells (Anywar 2020). The crude methanol root bark of *E. abyssinica* extracts was relatively safe with an LD_50_ of 776.2 mg/kg body weight in the acute toxicity tests on mice (Bunalema et al., 2012). The methanol, ethanol, ethyl acetate and diethyl ether extracts of *E. abyssinica* were not toxic to human keratinocyte cell line (HaCaT) IC_50_ > 512 (Shultz et al., 2020). The root bark of *E. abyssinica* contains alkaloids, tannins, terpenoids, saponins, flavones and phenols (Bunalema et al., 2012). In comparison, bioassay-guided fractionation *E. abyssinica* seeds by (Mohammed et al., 2012) yielded five alkaloids: erythraline, erysodine, erysotrine, 8-oxoerythraline and 11-methoxyerysodine. The relatively low cytoxicity *E. abyssinica* extracts coupled with the protective effect against HIV-1 induced cytopathogeneticy justify their use in traditional treatment of PLHIV.

#### *Gardenia ternifolia* Schumach. and Thonn.

*G. ternifolia* is traditionally used to treat opportunistic infections in PLHIV (Anywar et al., 2020a), malaria (Giday et al., 2009) and hypertension (Karou et al., 2011). It has proven *in vivo* antiplasmodial activity against *Plasmodium berghei* (Nureye et al., 2018). The bark extract was moderately cytotoxic to U87CD4CXCR4 cells with CC_50_ values of 53.0 and 42.3 μg/ml for the ethanol and DMSO extracts respectively (Anywar, 2020). (Tshitenge et al., 2017). found eight furan neolignans called gardenifolins A–H (1a–d and 2a–d), with varying levels of cytotoxicity’s against HeLa cell lines. The isomers, 1d and 2a particularly had the highest cytotoxities, IC_50_ = 21.0 and 32.5 μM, respectively. *Gardenifolin* D (1d) induced apoptosis of HeLa cells at 25 μM. There is no data on the ant-HIV activity of this plant species. Therefore, more research needs to be conducted to investigate its potential benefits in PLHIV.

#### *Gymnosporia senegalensis* (Lam.) Loes.

*Maytenus senegalensis* (synonym *Gymnosporia senegalensis*), is used for treating opportunistic infections in PLHIV (Anywar et al., 2020a), dyspepsia, wounds, chest pain, rheumatism and diarrhoea (Sosa et al., 2007). Both the DMSO and ethanol extracts of *G. senegalensis* were moderately toxic to U87 glioma cells U87CD4CXCR4 with CC_50_ values of 70.2 and 90.0 μg/ml respectively (Anywar 2020). The cytotoxicity of five chemotaxonomically significant compounds out of the 18 isolated compounds from the leaves of *M. senegalensis* were tested against colon (DLD1), breast (MCF7) and gastric (MKN45) cancer cells with weak to moderate decrease of viability at 50 μM (Tatsimo et al., 2019). A 70% ethanol extract of *M. senegalensis* had significant anti-inflammatory properties but was somewhat toxic in adult male CD-6 mice at 1200 mg/kg—ten times the anti-inflammatory dose (Sosa et al., 2007) *M. senegalensis* contains maytenonic acid (Tatsimo et al., 2019), β-sitosterol, coumarins such as scopoletin and prenyletin, alkaloids such as the pyrrolidine and stachydrine (da Silva et al., 2011), phenolic compounds, maytansinoids and maytenoic acid (Lindsey et al., 2006; Pistelli et al., 1998; da Silva et al., 2011). The relatively low cytoxicity *E. abyssinica* extracts coupled with the protective effect against HIV-1 induced cytopathogeneticy justify their use in traditional treatment of PLHIV.

#### *Psorospermum febrifugium* Spach.

Members of the genus *Psorospermum* have been used as febrifuges, purgatives and for the treatment of various skin conditions including leprosy, dermatitis, scabies, eczema, and subcutaneous wounds (Epifano et al., 2013). Members of this genus have also been reported to have antibacterial, anti-protozoal, anti-fungal, anti-viral, anticancer, anti-oxidant, and neuroprotective effects (Turrill and Milne-Redhead, 1952). *P. febrifugium* is widely used in treating opportunistic infections especially those that affect the skin (Anywar et al., 2020a). Extracts of *P. febrifugium* were tested against the following human tumoral cell lines with the corresponding IC_50_ values. Ovary carcinoma lines A2780, 2.63 μg/ml and A2780cis, 3.59 μg/ml, squamous epidermal carcinoma cell line A431, 1.61 μg/ml, and the melanoma cell line WM35 1.28 μg/ml, respectively (Hostettmann et al., 2000). The petroleum ether (ED_50_ = 0.35 μg/ml) and chloroform extracts (ED_50_ = 0.48 μg/ml of *P. febrifugium* root bark inhibited growth of the Co-115 colon carcinoma cell line (Hostettmann et al., 2000). The ethanol and DMSO extracts of *P. febrifugium* were moderately cytotoxic to U87CD4CXCR4 cells with CC_50_ values of 99.45 and 109.65 μg/ml for the ethanol and DMSO extracts respectively. The ethanol extracts of *P. febrifugium* exhibited significant *in vivo* cytotoxicity against P-388 lymphocytic leukaemia (3PS) in mice as well *in vitro* antitumor activity against a human derived epidermoid carcinoma cell culture of the nasopharynx (9KB) (Abou-shoer et al., 1989).

Psorospermin is a cytotoxic dihydrofuranoxanthone first isolated from the roots of *P. febrifugum* (Gebre-Mariam et al., 2006). Psorospermin showed significant activity against human colon adenocarcinoma (HT-29), human lung carcinoma (A-549), and human breast adenocarcinoma (MCF-7) cell lines (Cassady et al., 1990). Other compounds that have also been isolated from *P. febrifugum* include tetrahydroanthracene derivatives, vismione D, anthraquinone derivatives, 3-geranyloxyemodin, emodin and 2-geranylemodin (Botta et al., 1983; Botta et al., 1985; Cassady et al., 1990; Epifano et al., 2013), xanthonolignoids, isocadensin D, isocadensin monoacetate; cadensin D, F and G (Hostettmann et al., 2000), tetrahydrofurobenzofuranoxanthones, psorofebrin and hydroxyisopsorofebrin (Abou-Shoer et al., 1993) dihydrofuranoxanthone epoxide (Abou-Shoer et al., 1988), chrysophanic acid, 2-isoprenylemodin and ferruginin B (Both et al., 1983). Vismione D and its acetyl derivatives were found to be as active as 5-fluorouracil but less active than vinblastine against human colon carcinoma cell lines (Marston et al., 1986). Emodin from *P. febrifugum* has been identified as an antitumor compound for treating lung, prostate, ovarian, colon and hepatic cancers (Tamokou and Kuete, 2014). Research needs to be conducted on the specific anti-HIV effects of *P. febrifugum*, since its anticancer properties have been widely investigated.

#### *Securidaca longipedunculata* Fresen.

The root of *S. longipendunculata* is commonly and widely used in Africa for various aliments including sexually transmitted infections, coughs, fever, malaria and tuberculosis (Anywar et al., 2020a; Mongalo et al., 2015). The DMSO root bark extract (CC_50_ = 98.3 μg/ml) was moderately cytotoxic but the ethanol extract was weakly toxic or non-toxic (CC_50_ = 90.0 μg/ml) to U87CD4CXCR4 cells, suggesting that the root bark contains non-polar toxic substances (Anywar, 2020). Similarly, the last of seven fractions from the root extract of *S. longipendunculata* obtained with a gradient of methanol and chloroform significantly inhibited proliferation of U87 cell line (IC_50_ = 20.54 μg/ml) and induced apoptosis via cleavage of Poly-ADP-Ribose-Polymerase (PARP) (Ngulde et al., 2019). The chloroform root extract of *S. longipedunculata* was preferentially cytotoxic to PANC-1 human pancreatic cancer cells in nutrient-deprived medium. The aqueous root bark extract was toxic to Ehrlich ascites tumour cells with a mortality rate of 82.5% at 1000 μg/ml, and an IC_50_ = 67 μg/ml (Lawal et al., 2012). The methanol root extract of *S. longipendunculata* is relatively toxic to brine shrimp, LC_50_ = 77.1 μg/ml (Moshi et al., 2006) and LC_50_ = 74.18 μg/ml (Adiele et al., 2013). Adeyemi et al. (Adeyemi et al., 2010) evaluated the acute toxicity of the aqueous root extract of *S. longipendunculata* root extract in albino mice with LD_50_ of 1.74 g/kg and 0.02 g/kg when administered orally and intraperitoneally respectively.

The root of *S. longipedunculata* contains muchimangins E and F which are oxygenated xanthones, polymethoxylated xanthones, benzyl benzoate, benzophenones and benzoic acid derivatives (Dibwe et al., 2013; Dibwe et al., 2014) toxic saponins, and methyl salicylate, a suspected nephrotoxin which can cause renal damage (Watt and Breyer-Brandwijk, 1962; Colson and De Broe, 2005). The xanthones 1,6,8-trihydroxy-2,3,4,5-tetramethoxyxanthone and 1,6-dihydroxy-2,3,4,5,8-pentamethoxyxanthone contained in *S. longipedunculata* exhibited potent and selective cytotoxicity with potent activity values (PC_50_) of 22.8 and 17.4 μM, respectively (Dibwe et al., 2013). The specific anti-HIV effects of *S. longipedunculata* need to be investigated to ascertain its benefits in PLHIV.

#### *Vachellia hockii* (De Wild.) Seigler and Ebinger

Many species of *Acacia* are widely used across Africa in managing opportunistic infections in HIV/AIDS (Anywar et al., 2020a; Chinsembu and Hedimbi, 2010; Kisangau et al., 2007). *A. hockii*, a commonly used synonym of *Vachellia hockii* is used in the treatment of opportunistic infections in HIV/AIDS generally, but also anaemia, blood cleanser and cough (Anywar et al., 2020a) and hernia (Kibuuka and Anywar, 2015). The ethanol and DMSO extracts of *V. hockii* were moderately cytotoxic in U87.CD4.CXCR4 cells, with CC_50_ of 100.3 and 89.0 μg/ml respectively (Anywar, 2020). Acute oral toxicity tests using a single dose of 2000 mg/kg body weight of the aqueous extract of *V. hockii* in rats revealed no visual symptoms of toxicity or mortality during the observation period. Phytochemical analysis of the aqueous and ethanolic extracts of *V. hockii* showed the presence of alkaloids, flavonoids, anthracene derivatives, tannins, triterpenes, saponins and lignans in aqueous and coumarins (Lagnika et al., 2016). The toxicity and anti-HIV activity of *V. hockii* needs to be investigated in-depth to yield more information on its potential benefits.

#### *Warburgia ugandensis* Sprague.

*W. ugandensis* is one of the most commonly used multipurpose medicinal plant species in Uganda and is also widely used in other countries like Kenya, Tanzania and South Africa (Anywar et al., 2020a; Mwitari et al., 2013). The anti-HIV activity of *W. ugandensis* extracts was hindered by their high toxicity (Anywar, 2020). Mwitari and colleagues have shown *W. ugandensis* to be cytotoxic to intestinal epithelial cells IEC-6, with IC_50_ values < 50 μg/ml (Mwitari et al., 2013). Both the DMSO (CC_50_ = 1.5 μg/ml) and the ethanol (CC_50_ = 7.6 μg/ml) root were highly cytotoxic to U87CD4CXCR4 cells (Anywar, 2020). *W. ugandensis* contains cytotoxic sesquiterpenes called muzigadials (Olila et al., 2001). Anywar et al. (Anywar et al., 2020a) previously documented the careful and judicious use of *W. ugandensis* by experienced herbalists in Uganda *W. ugandensis* had CC_50_ > 250 μg/ml in Vero E6 cells and was classified as not cytotoxic. *W. ugandensis* aqueous stem bark extracts were also found to be non-toxic in BALB/c mice, with the LD_50_ > 5000 mg/kg body weight and no mortality recorded (Karani et al., 2013). However, when decoctions of *W. ugandensis* are used in doses exceeding what is prescribed by the herbalist, toxic effects such as vomiting, dizziness, weakness and ulcers are experienced. Because of its popularity and toxicity, the use of *W. ugandensis* should be with caution. Anywar et al. documented prolonged boiling of *W. ugandensis* decoctions for up to 6 h and administering it in small amounts by herbalists in Uganda (Anywar et al., 2020a). *W. ugandensis* extracts were contraindicated in pregnancy and in weak patients (Anywar et al., 2020a; Anywar, 2020). Like *A. coriaria*, there is conflicting information on the toxicity of *W. ugandensis*. Whereas one study indicated *W. ugandensi* to be cytotoxic, one study in mice and another cellular assay indicate that *W. ugandensis* is not toxic. Reports of toxicity by herbalists need to be further investigated in different cell-lines. The anti-HIV activity of *W. ugandensis* needs to be investigated to justify its popular use in HIV treatment.

#### *Zanthoxylum chalybeum* Engl.

*Z. chalybeum* is traditionally used for treating various opportunistic infections in HIV (Anywar et al., 2020a), malaria, sickle cell disease, measles, skin infections, jaundice, yellow fever and coughs (Olila et al., 2002; Kuglerova et al., 2011; Schultz et al., 2020; Schultz et al., 2020). The ethanol extracts of *Z. chalybeum* found was found to be weakly cytotoxic (CC_50_ = 231.0 μg/ml) whereas the DMSO extract was moderately cytotoxic (CC_50_ = 39.8 μg/ml) against U87CD4CXCR4 cells (Anywar, 2020). The methanol, ethanol, ethyl acetate and diethyl ether extracts of *Z. chalybeum* were not toxic to human keratinocyte cell line (HaCaT) IC_50_ > 512 (Schultz et al., 2020). The methanol extract of *Z. chalybeum* contains 9-Methoxychelerythrine, Sesamin (fagarol) and, nitidine (Addae-Kyereme et al., 2001). More research needs to be conducted on the anti-HIV activity and toxicity of *Z. chalybeum*.

### Use of Toxic Plant Species for Medicinal Purposes and Their Potential Benefits

The plant species under review are often used in combination with other herbs to reduce their toxicity. Skilled herbalists normally administer known toxic medicinal plants albeit in low doses and in polyherbal preparations (Anywar et al., 2020a). This can potentially neutralize the toxicity of some medicinal plant compounds. However, polyherbal preparations can also have unpredictable and complicated effects arising from the various interactions that can occur among the individual components (Che et al., 2013) and could increase the cytotoxic effect (Di Nunzio et al., 2017).

Many toxic herbs have been used traditionally to treat various ailments. The use of toxic herbs is acceptable or even desirable and preferred in the treatment of certain severe and critical conditions such as cancers (Wang et al., 2012). For instance, there are well-documented cases where plant species with toxic properties are used in Chinese traditional medicine, but are neutralised or detoxified with other herbal adjuncts. Thus, the therapeutic use of toxic herbs is not unusual, since toxicity can occur at varying levels of adverse or undesirable effects, which may not necessarily be lethal (Che et al., 2013). For example, *Zingiber officinale* (ginger) is used to neutralize the toxic effects of the tuber of *Pinellia ternata*, without compromising its therapeutic effects. *P. ternata* causes severe mucosal irritation and inflammation in the throat and the gastrointestinal tract, due to the presence of calcium oxalate and agglutinin (Wang et al., 2012; Zhu et al., 2012).

Whereas some plant extracts are known to be highly cytotoxic to particular cell types, cytotoxicity may be potentially beneficial as anticancer or anti-tumour drugs if they are selectively cytotoxic (Itharat et al., 2004; Zingue et al., 2018). The cytotoxicity of the plant extracts is desirous against cancer cells and researchers actively look out for selectively cytotoxic compounds against specific cancer cells, as potential drug candidates (Itharat et al., 2004).

### Toxicity in Primary Cells vis-à-vis Tumour Cell Line

Research has shown some plant extracts to be selectively cytotoxic to tumour cell lines in comparison to normal cells. For instance, Itharat et al. (Itharat et al., 2004) investigated the cytotoxicity of some medicinal plant species used by traditional doctors in Thailand to treat cancer patients using one normal cell line (the human keratinocytes SVK-14) and three human cancer cell lines: the large cell lung carcinoma COR-L23, the breast adenocarcinoma MCF-7 and the colon adenocarcinoma LS-174T. The rhizomes of *Dioscorea membranacea* were selectively and highly cytotoxic against the cancer cell lines (IC_50_ = 11.8, 12.0 and 23.2 μg/ml for COR-L23, LS-174T and MCF-7 cell lines respectively) with much lower cytotoxicity against the normal cell line used (IC_50_ = 71 μg/ml SVK-14 cells). A similar trend was exhibited by two other plant species *Dioscorea birmanica* and *Siphonodon celastrineus* in the same study. Zingue et al. (Zingue et al., 2018) also demonstrated the cytotoxicity of the ethanolic extract of *A. seyal* to be significantly less pronounced in the non-tumour cell lines, HUVEC and MRC-5 compared to other cancer cell lines MCF-7, MDA-MB-231, 4T1, SK-MEL-28, and SF-295 used. However, in some other cases where both tumour and normal cell lines were used to test plant extracts traditionally used in cancer treatment, the extracts have shown little selectivity for the cancer cells. This has raised fears about their safety and efficacy in traditional treatments (Ashidi et al., 2010).

### *In Vitro* Cytotoxicity as a Predictor of *In Vivo* Toxicity

There are conflicting reports about the possibility of extrapolating cytotoxicity data to *in vivo* toxicity. Several authors have demonstrated significant correlations between *in vitro* cytotoxicity assays and *in vivo* toxicity with successful predictions of systemic toxicological effects (Ekwall, 1983; Halle et al., 2003; Groothuis et al., 2015). However, there are several instances where there is no clear relationship between cytotoxicity *in vitro* and acute toxicity *in vivo* (Garle et al., 1994; Das, 2018). This may arise since the cells used are functionally remote from their tissues of origin, lowering their likelihood to accurately predict acute *in vivo* toxicities (Flint, 1990). Blaauboer (Blaauboer, 2002; Blaauboer, 2003) also asserts that the lack of knowledge on the biokinetic behaviour of compounds *in vivo* makes it impossible to directly use data derived from *in vitro* toxicity studies in assessing and extrapolating the toxicity of compounds in intact organisms.

### Future Needs and Priorities

Many of the medicinal plants used for treatment by PLHIV are used either in combination with other herbs or with antiretroviral drugs (ARV). This increases the chances of potentially harmful of herb-herb and herb-drug interactions. In addition, little is known about the toxicity of some of the plant species in use. Further studies need to be conducted to fully ascertain the toxicities of some plant extracts and determine any potential interactions of the medicinal plant extracts and their effects on the bioavailability of ARV. The toxicity of the plant extracts can also be investigated to determine if it is selective against cancer cell lines as potential anticancer agents.

## Conclusion

Most of the plant species reviewed apart from *P. febrifugium*, *S. longipedunculata* and *C. sanguinolenta* lack detailed phytochemical analyses involving the quantification and characterization of their chemical constituents. Even though crude plant extracts were the most commonly used, purified/single component extracts from different plant parts were also used in some studies. The U87 human glioblastoma cell line was the most commonly used cell type. Water, ethanol, methanol and DMSO were the commonest solvents used. In a few instances, isolated purified compounds/extracts such as Cryptolepine from *C. sanguinolenta* and Psorospermin from *P. febrifugium* were used for testing. The cytotoxicity levels varied with the type of cells, extracts and solvent used, making direct comparison of the plant species difficult. Five of the eleven plant species namely, *A. coriaria*, *C. sanguinolenta*, *G. ternifolia*, *P. febrifugium* and *Z. chalybeum* had no cytotoxicity studies in animal models reported. For the remaining six plant species, the crude aqueous and ethanol extracts were mainly in acute oral toxicity studies in mice. Only *A. coriaria* and *W. ugandensis* decoctions were reported to cause toxic side effects in humans by herbalists who administered them..
